# Cardiovascular Risk Is Increased in Miner’s Chronic Intermittent Hypobaric Hypoxia Exposure From 0 to 2,500 m?

**DOI:** 10.3389/fphys.2021.647976

**Published:** 2021-03-26

**Authors:** Andrés Pedreros-Lobos, Rodrigo Calderón-Jofré, Daniel Moraga, Fernando A. Moraga

**Affiliations:** ^1^Laboratorio de Fisiología, Hipoxia y Función Vascular, Departamento de Ciencias Biomédicas, Facultad de Medicina, Universidad Católica del Norte, Coquimbo, Chile; ^2^Departamento de Ciencias Básicas, Universidad Central La Serena, La Serena, Chile; ^3^Departamento de Medicina, Facultad de Ciencias de la Salud, Universidad de Tarapacá, Arica, Chile

**Keywords:** aerobic capacity, high sensitivity C reactive protein, corporal composition, cardiovascular risk, workers at high altitude, chronic intermittent hypobaric hypoxia

## Abstract

Over the past 40 years, mining activities in Chile have relocated miners who normally live at sea level to work at high altitudes. This results in a form of chronic intermittent hypobaric hypoxia (CIHH) characterized by alternating periods of work at high altitude and rest periods at sea level. Previous studies performed in our laboratory showed that aerobic capacity is reduced at 3,800 m, even when oxygen content is maintained. Our study aimed to determine the corporal composition, food intake, maximum oxygen uptake, and concentration of high sensitivity C reactive protein (*hs*CRP) in an acclimatized miner population that work from 0 to 2,500 m with CIHH exposure over 4 years. All miners recruited for our study were operators of heavy trucks with CIHH for over 4 years (shiftwork 7*7 days), and our experimental population was composed of 54 miners at sea level, 61 at 1,600 m, and 38 at 2,500 m. All evaluations were performed on the 3rd or 4th day of diurnal shiftwork. To determine corporal composition, we measured weight and height (to calculate body mass index, BMI), skinfolds (to calculate body fatty, BF), and waist circumference (WC); maximal aerobic capacity was evaluated using a ramp-incremental cycling to exhaustion protocol and a venous blood sample before the exercise test to measure (*hs*CRP) *via* an ELISA test. We found higher values of BMI, BF, and WC, in the miners’ population but observed no significant difference between populations. We found a decrease in VO_2_ of 11.6% at 1,600 m and 25.9% at 2,500 m compared to miners at sea level. An increase in (*hs*CRP) at 1,600 and 2,500 m regards sea level. We observed a high prevalence of overweight and obese subjects, which was related to the *ad libitum* availability of food and low physical activity (sedentarism). We found that work capacity was maintained despite a decreased VO_2_ max at moderate altitude. However, overweight and obesity support an increased risk of cardiometabolic disease in miner’s which is unrelated to altitude. In contrast, an increased *hs*CRP level could be associated with increased inflammatory mechanisms at 1,600 and 2,500 m.

## Introduction

Over the last 40 years, mining activity in Chile has relocated miners that normally live at low altitudes (<1,000 m) to work at high altitudes (>3,000 m). This shift is called the “Chilean model of Chronic Intermittent Hypobaric Hypoxia (CIHH) exposure,” which is characterized by alternating periods of work at high altitude and rest periods at low altitude (Richalet et al., 2002; Moraga et al., 2018).

Exposure to high altitude limits oxygen diffusion within the lungs, reducing oxygen transport into muscle and thereby affecting work capacity (Calbet and Lundby, 2009). However, exposure studies on maximal aerobic capacity in this model are scarce. Initial studies with CIHH miners began with a prospective study of CIHH exposure at a high altitude (4,500 m) for 31 months; where an inverse relationship between exposure time with decreased physical performance was observed (Richalet et al., 2002). A second study performed in soldiers with CIHH exposure for 6 months at a high altitude (3,550 m) showed a tendency for maximal aerobic capacity (VO_2_ max) to decrease at this altitude but oxygen transport capacity was maintained (Prommer et al., 2007). A third study performed on miners acclimatized to CIHH for a long period (7–36 months) showed that the maintained oxygen transport is explained by an increased hemoglobin concentration alongside increased intensity, reaching the same intensity (Watts) at sea level and high altitude (Moraga et al., 2018). Furthermore, a study performed in healthy miners, a population with moderate physical activity at high altitude, showed that work capacity (intensity) is maintained despite the reduced oxygen consumption, supporting the notion that increased work efficiency occurs during maximal exercise at high altitude (Moraga et al., 2019).

Several studies described an increasing prevalence of weight gain and obesity along with other cardiovascular risk factors in native highlanders (Shah et al., 2004). A series of antecedents showed that obesity not only predisposes people to insulin resistance and diabetes but also contributes to atherogenic dyslipidemia (see reviewed by Libby et al., 2002). Additionally, adipose tissue can also synthesize cytokines such as TNF-∝ and IL-6 (Yudkin et al., 1999) thus obesity itself promotes inflammation and potentiates atherogenesis.

Growing antecedents reported in the literature indicate that elevated circulating inflammatory markers, such as C-reactive protein (CRP), predict coronary events, stroke, and progression of peripheral disease independent of the severity of atherosclerotic or ischemic events (Pepys and Hirschfield, 2003). Additionally, two studies have previously shown increased levels of IL-6 and CRP at high altitude compared to controls by two different ascent types (passive or active) at 4,559 m (Hartmann et al., 2000; Bailey et al., 2004). Hypoxia at high altitudes could be considered a new inflammatory stimulus below the expected range for inflammatory diseases, such as acute mountain sickness or high-altitude pulmonary edema (HAPE; Bailey et al., 2004). We evaluate whether an increase in work efficiency could be related to increased cardiovascular risk in miners that work at sea level and acclimatized mine workers exposed to moderate CIHH at 1,600 and 2,500 m.

## Subjects, Materials, And Methods

A cross-sectional study and descriptive scope were carried out in 153 male miners with more than 4 years of experience. They undergo shift work characterized by 7 days of work followed by 7 days of rest. All miners work for the same company, which has operations at all three altitude levels (sea level, 1,600, and 2,500 m). Our population was composed of heavy truck operators where 54 miners worked at sea level, 61 at 1,600 m, and 38 at 2,500 m. Evaluations were performed on the 3rd and 4th day of diurnal shiftwork at sea level or high altitude. Protocols used in this study followed the International Ethical Guidelines (according to the Helsinki declaration) and were approved by the Ethics Committee of the Facultad de Medicina, Universidad Católica del Norte, Chile, and the Medical Director of the mining company. All volunteers were informed of the possible risks and discomfort involved before giving their signed consent to participate.

### Corporal Composition

Our anthropometric evaluation considered body weight (BW, kg) and height (cm) using a scale balance (SECA model 767) to calculate body mass index (BMI, BMI = kg/m^2^). Waist circumference (WC, cm) was measured using inextensible metric tape (SECA model 201). Body fat mass was determined by measuring four skinfolds: bicipital, tricipital, subscapular, and suprailiac (Lange skinfold, Cambridge, Maryland) percentage of adipose (Fatty body, %) was calculated using the protocol described by Durnin and Womersley (1974); and, finally, we calculated fat mass (FM, kg) as FM = (BW*FB)/100 and lean body mass (LBM, kg) as LBM = BW − FM. All anthropometrical measurements were made by the same evaluator. Table 1 shows the classifications of cardiovascular risk concerning BMI, WC, body fat (%), and LBM (kg; Bray, 1998; Jensen et al., 2013; Macek et al., 2020). After evaluation of corporal composition, we measured oxygen saturation (SpO2, %) and heart rate (HR, bpm) by pulse oximetry (model 7500FO Nonin) and systolic arterial pressure (SAP) and diastolic arterial pressure (DAP) using a cardiorespiratory monitor (model BM3, Bionet).

**Table 1:** Reference values and classification criteria of cardiovascular risk.

BMI (kg/m^2^)		
Low	<18.5	Jensen et al., 2013
Normal	18.5–24.9	
Overweight	>24.9 and <29.9	
Obesity	>30	
**BF (%)**		
Normal	>12 and <20	Bray 1998
Overweight	>20 and <25	
Obesity	>25	
**WC (cm)**		
Normal	<94	Macek et al., 2020
Elevated	>94	
***hs*CRP (mg/L)**		
Low	<1	Libby et al., 2002
Moderated	>1 and >3	
High	>3	

### Determination of the Energetic Balance

#### Evaluation of Dietary Intake

We assessed dietary intake with a standardized 24 h recall food survey (Olivares et al., 2007). The 24 h recall is a method employed to assess the type of food and the quantities consumed in the last 24 h. The nutritional contribution obtained from the survey allowed us to quantify the food intake and calculate the nutritional contribution of this food, which we then compared to a standardized table of recommended nutritional intake for the Chilean population (Zacarías et al., 2018). The dietary contribution values were expressed in Kilocalories (Kcal). All surveys were carried out by a nutritionist (AP-L) with experience in the use of this instrument.

#### Estimation of Energetic Expenditure

In our study, we used FAO/WHO/UNU equations (2001) to estimate the basal metabolic rest (BMR). We calculated total energetic expenditure (EET) using the following equation; EET = BMR × physical activity index. We defined a physical activity index using three levels according to lifestyle type which had persisted longer than their job occupation: sedentary (mean 1.55), moderate (mean 1.76), and active (mean 2.1; FAO/WHO/UNU, 2004; Westerterp, 2017). Physical activity in the workplace at sea level, 1,600, and 2,500 m was evaluated indirectly by using a IPAQs survey (International Physical Activity Questionnaire short; Ainsworth et al., 2000) in the morning of the 4th day, previous to the maximal aerobic capacity test.

### Measurement of *hs*CRP

We collected blood samples from the brachial vein before entering the shift (between 07:00 and 08:00 am) on day 3 at the indicated altitude. Blood samples were centrifuged immediately after collection (3,000 rpm for 20 min) and the plasma fraction was frozen at −80°C until analysis. To measure high sensitive CRP (*hs*CRP) levels, an ELISA test was performed in duplicate using a commercial kit for serum and plasma (Human CRP/CRP Quantikine ELISA Kit; catalog number: DCRP00; R&D Systems, Minneapolis, MN 55413). Table 1 shows the CV risk criteria according to the plasma level of *hs*CRP.

#### Maximal Aerobic Capacity

The exercise test was performed on the 4th day on a cycle ergometer (Model Corival, Lode) where oxygen consumption and ventilation (VE) variables were measured using a metabolic cart (Ultima CPX, Medgraphics, St. Paul, Minnesota, United States) calibrated before each test according to the manufacturer’s instructions with high-grade calibration gases (purchased to INDURA, Chile). Respiratory variables were analyzed breath-by-breath in real-time and averaged 5 s during all tests. We also assessed transcutaneous arterial saturation (SpO2, %) and HR (bpm) by a pulse oximeter (7500FO Nonin Medical, Inc., United States) with the sensor placed on an ear lobe (8000Q2 Nonin Medical, Inc., United States). To measure maximum oxygen consumption (VO_2_ max), we performed a ramp-incremental cycling to exhaustion protocol followed by a 10-min rest period seated on the ergometer. Each participant was instructed to begin cycling at 0 Watts, maintaining a cadence of 70 rpm. The work rate was increased by 0.5 Watts/s (or equivalent to 30 Watts/min) thereafter until the participant reached voluntary exhaustion. We considered VO_2_ max when participants reached values over 85% of the estimated max load.

### Statistical Analysis

Values presented in tables were expressed as the mean ± SD. Non-parametrical variables obtained in our study, such as percentages (%) were analyzed by proportional tests. Comparison of parametrical variables was analyzed using one-way ANOVA, and differences between different altitudes were evaluated by a Newman-Keuls test. Differences were considered statistically significant when values of *p* < 0.05. Pearson’s correlation was also performed; the analysis was carried out to establish the association between *hs*CRP (dependent variable) with anthropometrical and cardiometabolic variables. All statistical analyses were performed with GraphPad Prism Software (version 5.03, GraphPad Software, Inc.).

## Results

### Corporal Composition

Populations studied here showed no variation in BW, BMI, body fatty (BF), FM, LBM, and waist perimeter between the levels evaluated (Table 2). However, when analyzing populations based on the established criteria (Table 1), we observed BMI values in the overweight and obese range at sea level, 1,600, and 2,500 m of 80, 75, and 84%, respectively, and BF (%) values of 57.5, 57.5, and 58.5%, respectively. Additionally, we observed WC measurements which corresponded to a high risk of cardiovascular disease (over 94 cm) at sea level, 1,600, and 2,500 m of 43, 48.6, and 52.9%, respectively.

**Table 2:** Anthropometrical characteristic of population that work at several altitudes.

	Altitude (m)		
	Sea level	1,600	2,500
*n*	54	61	38
Ages (years)	42.5 ± 10.5	37.2 ± 8.0	37.7 ± 9.1
Weight (kg)	78.3 ± 11.4	83.2 ± 11.2	81.2 ± 10.6
Height (m)	1.70 ± 0.07	1.73 ± 0.06	1.70 ± 0.05
BMI (kg/m^2^)	27.2 ± 3.1	27.7 ± 2.7	28.1 ± 2.8
BF (%)	25.5 ± 4.2	26.9 ± 4.3	27.5 ± 4.4
BFM (kg)	21.7 ± 5.7	22.5 ± 5.3	21.8 ± 5.6
LBM (kg)	56.7 ± 7.0	60.7 ± 7.8	59.4 ± 6.5
WC (cm)	94.5 ± 8.9	95.1 ± 7.6	95.0 ± 8.0
**Physical activity (%)**			
Active	14.6	2.7	7.5
Moderate	17.1	21.6	24.0
Sedentary	68.3	75.7	68.5

*Mean ± SD, Body mass index (BMI), Body fatty (BF), Body fatty mass (BFM), Lean body mass (LBM), and Waist circumference (WC)*.

### Energetic Balance at Several Altitudes

#### Dietary Intake

The body composition results described above show that the population has risk factors associated with overweight and obesity, due to the dietary intake of volunteers being above normal dietary intake levels (Table 3). The population we studied also lived a largely sedentary lifestyle. We observed sedentarism rates at sea level, 1,600, and 2,500 m of 68, 75, and 68%, respectively, which is consistent with previously described levels of overweight and obese people. To estimate EET, we considered 1.55 as a cut off factor for physical activity at all three altitude levels (Table 3) and observed that subjects with an average EET above 110% at sea level, 1,600, and 2,500 m was 67, 58.8, and 45%, respectively. EET above 110% would tend to promote weight gain, therefore, the higher the energy balance percentage, the greater the risk of developing overweight or obesity.

**Table 3:** Energetic balance in population that work at several altitudes.

	Altitude (m)		
	Sea level	1,600	2,500
Intake (Kcal/day)	2,305 + 232	2,341 + 512	2,940 + 409^*^ ^†^
Lost (Kcal/day)	2,102 + 154	2,229 + 192^*^	2,507 + 231^*^
Energetic balance	110	105	117
Energetic balance (<110%)	65.9	51.9	58.3

* Mean ± SD vs. sea level.

† Mean ± SD vs. 1,600 m (p < 0.05).

### *Cardiorespiratory Variables and hs*CRP Plasma Concentration at Sea Level, 1,600, and 2,500 m

**Figure 1:**
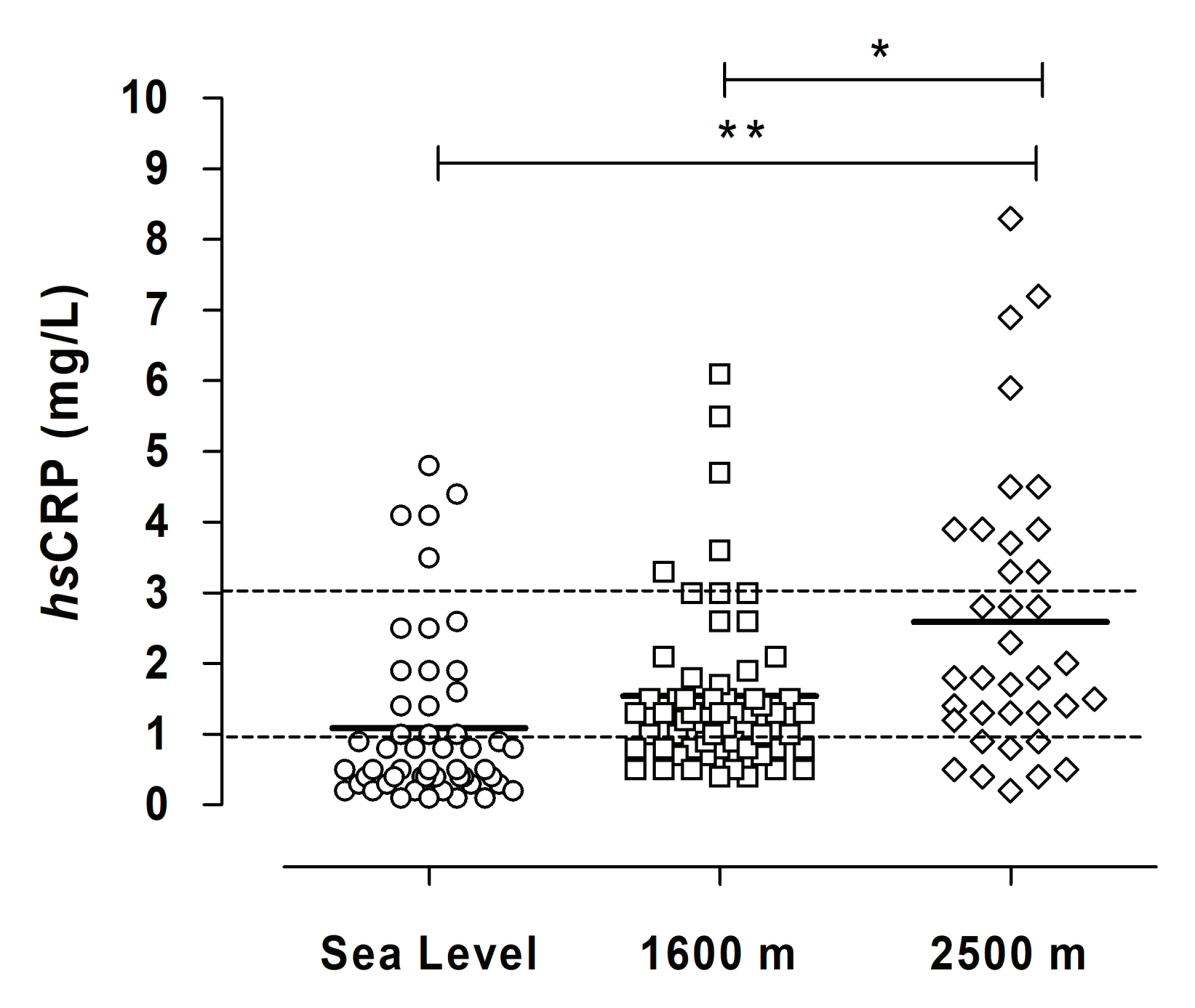
Plasma concentration of *hs*CRP in miners exposed to CIHH at several altitudes. The horizontal thick line represents a mean value of *hs*CRP at each altitude. The horizontal dotted line represents a cut-off of cardiovascular risk (<1 mg/L, low; >1 and <3, moderate; and >3 high). Asterisks represent a significant difference between sea level and 2,500 m (*p* < 0.05) and double asterisks represent a significant difference between 1,600 and 2,500 m (*p* < 0.05).

We performed cardiorespiratory evaluations at each altitude and observed a progressive decline in arterial oxygenation with increasing altitude (Table 4). Conversely, cardiovascular variables such as systolic and diastolic blood pressure and HR increased with altitude (Table 4). Additionally, we observed that *hs*-CRP levels in plasma significantly increased at 2,500 m, compared to sea level and 1,600 m (Figure 1), and we observed a significant increase in the percentage of subjects with values over 3 mg/L (which indicates an increased risk of cardiovascular disease, see Table 1); 33.3% at 2,500 m compared with 9.6 and 8.5% at sea level and 1,600 m, respectively. Supplementary Table shows the correlation between *hs*CRP vs. anthropometrical and cardiometabolic variables, where we observed a positive and significant correlation with SAP and a negative and significant correlation with VO_2_ max (expressed mlO2/min/kg and mlO2/min/kg LBM) at 2,500 m. Additionally, a positive and significant correlation was observed with BF, LBM, and WC and a negative and significant correlation was observed with VO_2_ max (mlO2/min/kg).

**Table 4:** Basal cardiorespiratory evaluated in population that work at several altitudes.

	Altitude (m)		
	Sea level	1,600	2,500
Oxygen saturation (%)	98.5 ± 0.9	94.0 ± 1.1^*^	92.8 ± 1.3^*^^†^
Heart rate (bpm)	70.4 ± 8	75.1 ± 11.5	78.6 ± 10.9^*^
Systolic arterial pressure (mmHg)	127.8 ± 10.6	131.7 ± 11.3^*^	125.5 ± 8.8
Dyastolic arterial pressure (mmHg)	77.2 ± 9.3	86.5 ± 8.2^*^	83.3 ± 7.9

* *Mean* ± *SD vs. sea level.*

† *Mean* ± *SD vs. 1,600 m (p < 0.05).*

### Maximum Oxygen Consumption (VO_2_ max)

Resting and maximum values of cardiorespiratory and metabolic parameters at sea level, 1,600, and 2,500 m are shown in Table 5. We observed a significant difference between resting and maximum exercise in VO_2_, SpO2, HR, VE, and RER (*p* < 0.05). Additionally, when we compared cardiorespiratory and metabolic responses obtained at rest at sea level and high altitude, we observed a significant decline in oxygen saturation at 1,600 and 2,500 m (*p* < 0.05) and a significant increase in HR at 2,500 m (*p* < 0.05). No difference was observed in VO_2_, VE, and RER (*p* > 0.05). However, when we compared cardiorespiratory and metabolic responses obtained at maximum exercise, we observed a progressive decline in maximum oxygen consumption (expressed mlO2/min/kg) at 2,500 m (*p* < 0.05). We observed a similar pattern when comparing maximum oxygen consumption and LBM. These low VO_2_ max levels were obtained at the same intensity (Watts), RER, VE, and HR at sea level, 1,600, and 2,500 m. However, a gradual decrease in oxygen saturation was observed during maximum exercise at 1,600 and 2,500 m (*p* < 0.05).

**Table 5:** Resume of cardiorespiratory variables evaluated at maximum exercise that work at several altitudes.

	Altitude (m)		
	Sea level	1,600	2,500
**Intensity (Watts)**			
Rest	0	0	0
Maximum	174 ± 33	198 ± 25	173 ± 27
**VO_2_ (mlO_2_/kg/min)**			
Rest	3.6 ± 0.7	3.5 ± 0.9	3.1 ± 0.8
Maximum	25.9 ± 5.8^†^	22.9 ± 4.8^†^	19.2 ± 5.3^*^ ^†^
**VO_2_ (mlO_2_/kg (BLM)/min)**			
Rest	5.0 ± 1.0	4.8 ± 1.2	4.2 ± 1.1
Maximum	36.1 ± 6.2^†^	31.2 ± 5.1^†^	26.6 ± 6.2^*^ ^†^
**RER**			
Rest	0.84 ± 0.09	0.90 ± 0.10	0.86 ± 0.07
Maximum	1.20 ± 0.19^†^	1.27 ± 0.15^†^	1.23 ± 0.17^†^
**VE (L/min)**			
Rest	8.9 ± 2.4	9.4 ± 3.1	9.6 ± 2.2
Maximum	65.6 ± 17.2^†^	79.3 ± 18.8^†^	69.3 ± 18.4^†^
**Oxygen saturation (SpO2, %)**			
Rest	97.5 ± 0.5	94.5 ± 1.8^*^	92.8 ± 1.2^*^ ^‡^
Maximum	93.2 ± 1.1^†^	90.2 ± 1.7^*^ ^†^	88.3 ± 1.9^*^ ^†^
**HR (bpm)**			
Rest	71 ± 11	76 ± 10	80 ± 12^*^
Maximum	151 ± 16^†^	164 ± 14^†^	154 ± 16^†^

* Mean ± SD vs. sea level (p < 0.05).

† Mean ± SD vs. rest (p < 0.05).

‡ *Mean ± SD vs. 1,600 m*.

*Heart rate (HR), Pulse oximetry (SpO2), and Ventilation (VE)*.

## Discussion

Our results show that miners who work at moderate altitudes must maintain a similar workload, VE, and HR with decreased oxygen saturation and VO_2_ max, and therefore must endure a greater cardiovascular challenge than those at sea level. Additionally, we observed an increased prevalence of sedentarism and overweight and obesity in all groups, and moderate altitude resulted in increased *hs*-CRP levels.

### Corporal Composition and Nutrition

Our study shows that the average anthropometric values (BMI, BF, MF, WC) in the miners were higher than the national average according to the 2010 National Health Survey (Ministry of Health Chile, n.d.). Similar results were described in a Chilean miner population with CIHH exposure (Pedreros et al., 2018) who presented higher obesity and overweight rates. Additionally, increased blood pressure, cholesterol, and glucose levels were observed in workers of the mining industry exposed to intermittent high-altitude hypoxia at 3,700–4,000 m in the Kyrgyz Republic (Esenamanova et al., 2014). However, in a prospective study performed in Chilean miners without previous exposure to high altitude (3,800–4,600 m), it was shown that BW and body composition did not change significantly after 31-month (Richalet et al., 2002). The anthropometric information we collected in the present study is contradictory to that indicated in the literature. In the literature, it has been described that exposure to altitude under an acute exposure model leads to weight loss, due to a negative energy balance mainly caused by an increased basal metabolic rate and suppression of appetite (Lippl et al., 2010). Some studies have even shown that staying in a hypobaric hypoxic environment could even be used as a treatment for obesity due to the reasons noted above (Lippl et al., 2010; Palmer and Clegg, 2014; Karl et al., 2018). However, regarding our result, we do not observe that this positive effect translates into a reduction in overweight and obesity in our population. We believe that our results are due to lifestyles where energy intake exceeds expenditure (Vearrier and Greenberg, 2011), which could be partially explained by cultural aspects, such as the mining organizational structure, workers union pressure, and mining worker food-culture. A study published on miners with cardiovascular risk factors showed that the daily energy intake that a worker consumes could reach 6,378 Kcal/day, and was largely made up of energy-dense foods rich in simple sugars, sodium, cholesterol, saturated fatty acids, and with a low fiber content (Padilla, 1999). However, another study classified miner’s physical activities as low in administrative workers (estimation of 2.2–2.4 Kcal/day), moderate in truck operators (estimation of 2.4–2.6 Kcal/day), and active in mechanical workers (estimated 2.8–3.0 Kcal/day; Caichac et al., 2013). In our study, we considered the population represented by truck operators to have a physical activity rate as light or sedentary.

### Cardiovascular Effects

High altitude is associated with increased sympathetic tone and may result in elevated blood pressure (Richalet et al., 1992; Naeije, 2010). In a previous study, the authors measured arterial blood pressure for 24 h in miners at sea level and high altitude (3,800 m) and found that the mean arterial pressure at high altitude was higher than at sea level, supporting the notion that high altitude leads to increased sympathetic tone (Richalet et al., 2002). Additionally, the authors found a reduction in mean arterial pressure after 31 months of exposure to CIHH. A similar response was described in the miner’s population exposed to CIHH at 4,000 m (Kyrgyzstan; Vinnikov et al., 2016). In contrast, in another miner population exposed to CIHH at 3,700–4,000 m, high overweight and obesity rates are prevalent and associated with increased blood pressure (Esenamanova et al., 2014). However, in our study, we did not observe any changes and /or presence of subjects with elevated arterial pressure in the population exposed to sea level compared to 1,600 or 2,500 m.

We observed a significant increase in *hs*CRP plasma concentration at 1,600 and 2,500 m in miners exposed to CIHH. It was previously shown that non-specific inflammation could be induced by hypoxia and contribute to high altitude-associated diseases. Three studies have found increased levels of IL-6 and CRP at high altitude compared to baseline levels during both passive or active ascent of Capanna Regina Margherita (4,559 m). IL-6 peaked on the second day and declined to baseline during the following 3 days. Additionally, CRP levels increased on day 3 and remained elevated before descending (Hartmann et al., 2000; Bailey et al., 2004). A study performed on subjects without previous prolonged exposure to high altitude showed that these people presented increased plasma *hs*CRP levels when they stayed at 4,000 m for 3 months (Hu et al., 2016). In these studies, the authors proposed that hypoxia at high altitudes could be considered a new inflammatory stimulus below the range expected for inflammatory diseases, such as acute mountain sickness or HAPE. Early studies describe a high increase in IL-6 and CRP levels with HAPE in subjects at 4,559 m (Kleger et al., 1996). However, no miner exposed to CIHH at 4,500 m suffered from severe forms of mountain sickness (HAPE or HACE; Richalet et al., 2002) and an incidence of HAPE 0.49% and no coronary events were observed during the construction of the Qinghai-Tibet railroad (Wu et al., 2007). Also, in our study, we did not observe HAPE at 1,600 and 2,500 m. The higher *hs*CRP values in miners could also be explained by the high prevalence of overweight and obesity levels previously described: since obesity (excess adipose tissue) is characterized by a state of permanent mild inflammation with increased circulating levels of inflammatory markers such as *hs*CRP, IL-6, TNF∝, and others (more detail see Kayser and Verges, 2013). However, the high prevalence of overweight and obesity described in the present study is not related to altitude, suggesting that the observed increase is the result of altitude exposure. A study performed in Puno-Perú (3,825 m) show elevated values of *hs*CRP (>3 mg/L) in individuals with lower values of oxygen saturation (Miele et al., 2016). Also, lower resting daytime oxygen saturation may serve as a marker of increased cardiovascular risk at high altitudes (Grundy et al., 2004).

### Maximal Oxygen Consumption at High Altitude

Many studies have reported the fall in VO_2_ max at high altitudes (see West, 2006). A study performed by Cerretelli (1980) reports that this fall is consistent with acute or chronic exposure and Kayser (2005) reported a VO_2_ maximum decrease by 1% for every additional 100 m elevation over 1,500 m. Therefore, VO_2_ max could decrease by 1–2% at 1,600 m and a reduction of 10% in the VO_2_ max at 2,500. However, our results showed a reduction of 11.6% of VO_2_ max at 1,600 m and 25.9% at 2,500 m, a difference of nearly 10% at 1,600 m and 15.9% at 2,500 m. We previously described a 29% reduction in VO_2_ max in miners exposed CIHH at 3,800 m. This reduction was similar to that described by other authors associated with a decreased intensity (Sutton et al., 1988). However, in our previous studies, this fall in VO_2_ max does not correlate with a fall in intensity and was interpreted as an increase in work efficiency at 3,800 m (Moraga et al., 2018, 2019) due to a major effort in the respiratory muscle work (see discussion, Moraga et al., 2019). In contrast, we reported a reduction of 25.9% in the VO_2_ max at 2,500 m where the intensity of exercise, VE, and HR was lower than that described at 3,800 m (Moraga et al., 2019). A possible explanation for the lower aerobic capacity in our study population could be a worse physical condition, since over 70% of subjects were sedentary, overweight, and aged. This worse physical condition could be corroborated by values of maximal effort (extenuating) at the lower value of 200 Watts at 1,600 and 2,500 m and lower HR values. Similarly, a decreased aerobic capacity in miners was previously reported at an intensity of 175 W, representing a decreased maximal intensity of ~15% after 31 months of CIHH exposure in subjects evaluated at sea level (Richalet et al., 2002). Reduced HR was also explained by the downregulation of β-adrenergic receptors, upregulation of muscarinic receptors (Richalet et al., 1992), and a detraining effect of exposure to hypoxia and/or being excessively sedentary (Richalet et al., 2002). We considered that the lower VO_2_ max values we observed in the present study could be due to a significant FM. However, when we calculated VO_2_ max/LBM (kg), values of VO_2_ max were enhanced but the percentage of change in VO_2_ max at an altitude of 1,600 and 2,500 m compared to sea level was maintained (Table 4). This evidence suggests that the fall in VO_2_ max is due to a lower aerobic capacity as a result of sedentarism, rather than an increase in the fatty mass in our population.

## Limitations

We recognize a series of limitations in our assessment of miners who work at sea level or a geographic altitude of 1,600 and 2,500 m. For instance, it was impossible to evaluate the effect of exposure to high altitude on cardiorespiratory variables during the VO_2_ max test because unions opposed the use of rest time (descending) for evaluation. Our study did not evaluate other metabolic and cardiovascular biomarkers such as oxidative stress and/or antioxidant mechanisms. Gender equity studies in this population are very difficult since women workers at altitude (low, moderate, and high) are very low or anecdotal. However, the population of women that work at altitude is associated primarily with functional services (health, feeding, cleaning, and others). In future studies, we will consider monitoring arterial blood pressure for 24 h and evaluate other markers of endothelium dysfunction (NO, ADMA, Homocysteine) to obtain a better understanding of the mechanisms underlying the high-altitude physiological adjustments in this population of shift workers.

### Conclusion

We found that work capacity, HR, and VE with a decreased VO_2_ max is maintained at moderate altitude, suggesting that work efficiency is maintained. Higher prevalence of overweight-obesity, BF% and WC, and sedentarism in all miner populations are evidence of cardiometabolic risk that is not related to altitude. However, increased *hs*CRP levels were associated with altitudes of 1,600 and 2,500 m.

## Data Availability Statement

The original contributions presented in the study are included in the article/Supplementary Material, further inquiries can be directed to the corresponding author.

## Ethics Statement

The studies involving human participants were reviewed and approved by Ethics Committee of the Facultad de Medicina, Universidad Católica del Norte, Chile. The patients/participants provided their written informed consent to participate in this study.

## Author Contributions

FM conceived and designed the study. RC-J and AP-L supervised the overall study. RC-J performed the statistical analysis. DM, RC-J, and AP-L contributed to sample and data collections. All authors drafted the report and contributed to the interpretation of the results, critical revision of the manuscript, and approval of the final manuscript. FM is the guarantor.

### Conflict of Interest

The authors declare that the research was conducted in the absence of any commercial or financial relationships that could be construed as a potential conflict of interest.
