# Supplementary material for: Cardiovascular Risk Is Increased in Miner’s Chronic Intermittent Hypobaric Hypoxia Exposure From 0 to 2,500 m?
Source: Front Physiol. 2021 Mar 26;12:647976. doi: 10.3389/fphys.2021.647976 (PMC8032972; doi:10.3389/fphys.2021.647976)
Supplement: Supplementary file 1 [file Table_1.docx]

Supplementary table. Pearson’s correlation between *hs*CRP vs anthropometrical and cardiometabolic variables at sea level, 1600 m and 2500 m.

|  | Altitude (meters) | | | | | |
| --- | --- | --- | --- | --- | --- | --- |
|  | Sea Level | | 1600 | | 2500 | |
|  | r | p | r | p | r | p |
| BMI (Kg/m^2^) | -0.2338 | ns | +0.3477 | **0.022** | +0.2432 | ns |
| WC (cm) | -01767 | ns | -0.1335 | ns | +0.2980 | ns |
| BFM(Kg) | -0.2304 | ns | +0.5506 | **0.004** | +0.1897 | ns |
| LBM (Kg) | -0,1169 | ns | -0.0261 | ns | +0.2549 | ns |
| SpO2 (%) | 0.0172 | ns | -0.0302 | ns | -0.0117 | ns |
| HR (lpm) | 0.0541 | ns | +0.0616 | ns | +0.1730 | ns |
| SAP (mmHg) | -0.2513 | ns | -0.1335 | ns | +0.3935 | **0.007** |
| DAP (mmHg) | -0.2159 | ns | +0.1070 | ns | +0.2756 | ns |
| VO2(mlO2/min/ Kg) | -0.0082 | ns | -0.3325 | **0.031** | -0.4161 | **0.007** |
| VO2(mlO2/min/LBM Kg) | -0.0094 | ns | -0.2089 | ns | -0.3325 | **0.016** |
| Pearson’s correlation (r), values significance (p), Body mass index (BMI), Body fatty mass (BFM), Lean body mass (LBM), Waist circumference (WC), Pulse oximetry (SpO2), Heart rate (HR), Systolic arterial pressure (SAP), Diastolic arterial pressure (DAP), and Maximal oxygen consumption (VO2). | | | | | | |
